# Identification of Potential Kinase Inhibitors within the PI3K/AKT Pathway of *Leishmania* Species

**DOI:** 10.3390/biom11071037

**Published:** 2021-07-16

**Authors:** Rodrigo Ochoa, Amaya Ortega-Pajares, Florencia A. Castello, Federico Serral, Darío Fernández Do Porto, Janny A. Villa-Pulgarin, Rubén E. Varela-M, Carlos Muskus

**Affiliations:** 1 Programa de Estudio y Control de Enfermedades Tropicales PECET, Faculty of Medicine, University of Antioquia, Medellín 050010, Colombia; carlos.muskus@udea.edu.co; 2 Biophysics of Tropical Diseases Max Planck Tandem Group, University of Antioquia, Medellín 050010, Colombia; 3 Department of Medicine, The Peter Doherty Institute, University of Melbourne, Melbourne, VIC 3000, Australia; amayao@student.unimelb.edu.au; 4 Instituto de Química Biológica de la Facultad de Ciencias Exactas y Naturales (IQUIBICEN), IC-CONICET Ciudad Universitaria, Pabellon 2, Ciudad de Buenos Aires C1428EHA, Argentina; florencia.castelloz@gmail.com (F.A.C.); fedeserral92@gmail.com (F.S.); dariofd@gmail.com (D.F.D.P.); 5 Departamento de Química Biológica, Facultad de Ciencias Exactas y Naturales, Universidad de Buenos Aires Ciudad Universitaria, Pabellon 2, Ciudad de Buenos Aires C1428EHA, Argentina; 6 Grupo de Investigaciones Biomédicas, Facultad de Ciencias de la Salud, Corporación Universitaria Remington, Medellín 050034, Colombia; janny.villa@uniremington.edu.co; 7 Grupo de Investigación en Química y Biotecnología (QUIBIO), Facultad de Ciencias Básicas, Universidad Santiago de Cali, Cali 760035, Colombia

**Keywords:** kinases, bioinformatics, drug discovery, molecular docking, PI3K/AKT pathway

## Abstract

Leishmaniasis is a public health disease that requires the development of more effective treatments and the identification of novel molecular targets. Since blocking the PI3K/AKT pathway has been successfully studied as an effective anticancer strategy for decades, we examined whether the same approach would also be feasible in *Leishmania* due to their high amount and diverse set of annotated proteins. Here, we used a best reciprocal hits protocol to identify potential protein kinase homologues in an annotated human PI3K/AKT pathway. We calculated their ligandibility based on available bioactivity data of the reported homologues and modelled their 3D structures to estimate the druggability of their binding pockets. The models were used to run a virtual screening method with molecular docking. We found and studied five protein kinases in five different *Leishmania* species, which are AKT, CDK, AMPK, mTOR and GSK3 homologues from the studied pathways. The compounds found for different enzymes and species were analysed and suggested as starting point scaffolds for the design of inhibitors. We studied the kinases’ participation in protein–protein interaction networks, and the potential deleterious effects, if inhibited, were supported with the literature. In the case of *Leishmania* GSK3, an inhibitor of its human counterpart, prioritized by our method, was validated in vitro to test its anti-*Leishmania* activity and indirectly infer the presence of the enzyme in the parasite. The analysis contributes to improving the knowledge about the presence of similar signalling pathways in *Leishmania*, as well as the discovery of compounds acting against any of these kinases as potential molecular targets in the parasite.

## 1. Introduction

Leishmaniasis is a disease of poverty, affecting socially and economically disadvantaged populations living in poor hygiene and health conditions [1]. Caused by protozoan parasites from the *Leishmania* species, it is considered a group of diseases that range from disfiguring skin lesions to a potentially fatal, generalized visceral infection. An estimated 1 to 1.6 million new cases are reported each year worldwide in 99 countries throughout Africa, the Indian subcontinent, Latin America, the Middle East and the Mediterranean region [2].

Since no vaccines are available for human leishmaniasis [3,4], chemotherapy is one option for treatment. For more than 70 years, pentavalent antimonials have been the treatment of choice, while pentamidine [5], paromomycin [6], liposomal amphotericin B [7] and miltefosine [8] are chosen as second-line drugs. In any case, leishmaniasis requires long-term therapy, which is limited by the need of health personnel, progressive loss of efficacy, severe side effects and high economic costs, restricting patient adherence and inducing drug resistance [9].

One approach to tackle this problem consists of defining novel molecular drug targets within the parasite that can be associated with essential signalling cascades, using the information available in large biological databases [10,11,12]. This facilitates the identification of regulatory and effector molecules essential in parasite biology. Here, we focus on the study of *Leishmania* proteins predicted as kinases based on similarities with known proteins of the phosphoinositide 3-kinase (PI3K)/protein kinase B (AKT) pathway.

PI3K/AKT appears to be important for parasite cell cycle progression, proliferation and survival [13]. In other eukaryotes, it has been found that the deregulation of PI3K/AKT reduces cell proliferation, inducing cell cycle arrest and apoptosis. This phenomenon has largely been explored in the field of human health, and it is thought to be present in trypanosomatids [14]. The pathway is also a tightly regulated process with some opposite but supplementary roles to cell proliferation [15]. Morphological and biochemical features (as defined by the Nomenclature Committee on Cell Death, NCCD) appear to be conserved in *Leishmania* [16,17,18]. This pathway would be necessary to control cell growth when there is either a nutrient deficiency (shortage) to avoid host death or to inhibit the inflammatory response [19]. This motivates the identification of specific molecular targets within the pathway.

The structural information of these targets is crucial to perform computational screening of novel compounds or for the repurposing of known drugs to inhibit or modulate their activity. Methodologies such as molecular docking allow a rapid screening of potential candidates based on previous knowledge of the protein binding sites and active conformations [20]. Moreover, hybrid methodologies are available to include the protein flexibility in an indirect way using a molecular dynamics simulation of the target previous to the screening [21]. These methods have been applied in previous projects using the model of an AKT-like kinase of *Leishmania* and *Trypanosoma* [22,23], where a list of molecules were evaluated experimentally with promising results [24].

In this paper, we focused on the virtual screening of compounds able to modulate a set of kinases with potential orthologues on the PI3K/AKT pathway. For this purpose, we matched *Leishmania* proteins with human annotated kinases involved in the pathway. Then, a ligandibility metric was calculated with bioactivity data of the reported kinases, which were associated with the *Leishmania* proteins used in the screening. Structural models were built, and druggability metrics were calculated for their binding pockets. A virtual screening approach using molecular docking and a complementary ligand-based methodology was run to identify a pool of candidates. The chemical structures of the compounds were clustered and ranked based on their predicted scores and targets covered during the screenings. A known human GSK3 inhibitor found by our approach was tested experimentally against the parasite to validate its anti-*Leishmania* activity. To complement the study, the selected kinases were mapped to a protein–protein interaction network previously built for different *Leishmania* species to review their interactions [25], together with a systematic search on the literature to determine if they had been validated as therapeutic molecular targets in leishmaniasis, cancer or other human diseases.

## 2. Results and Discussion

### 2.1. List of Kinases Detected in Different Leishmania Species

According to the PI3K/AKT pathway reported in human, a list of kinases annotated in different species of *Leishmania* were mapped to human proteins across the pathway. The complete alignment results containing the kinases and other proteins are available in the Supplementary Table S1. The selected proteins were characterized by identity percentages between 40% to 70% with E-values < 1 × $10^{-10}$. The list of mapped *Leishmania* kinases is described in Table 1.

From Table 1, five proteins have been detected and officially annotated with kinase domains in all the *Leishmania* genomes. We included in the analysis other proteins participating in the pathways, but we found that a representative set is not detected in the parasite, providing some gaps in the explanation of how these routes are expressed or which possible mechanisms are used by *Leishmania* for exploiting the host protein machinery. The identification was also supported by a deep search in the literature regarding their participation in cell proliferation, survival and apoptosis-like routes within different *Leishmania* species.

### 2.2. Modelling, Ligandibility and Druggability Prediction

To assess if the selected can be studied as molecular targets, the ligandibility of their reported homologues was calculated. As shown in Table 2, all the kinases have values over 0.5, which indicates a higher success rate in the screening of novel ligands. Next, given that most kinases do not have experimental structures deposited in the PDB, we used homology-based models of the selected kinases to classify them according to their structural druggability.

**Figure 1:**
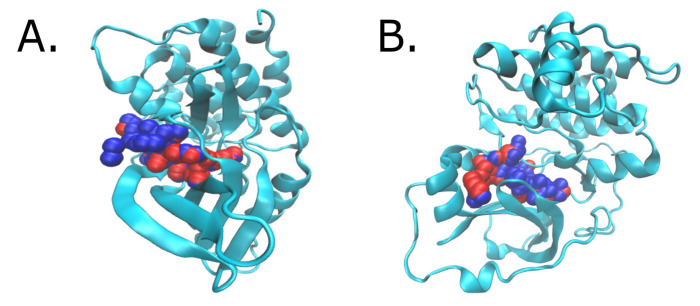
Protein structure visualization with VMD software for CDK (**A**) and AKT (**B**) homologues in *L. braziliensis*. The most druggable pocket is shown. Polar alpha spheres are depicted in red, while apolar alpha spheres are in blue.

The prediction of the pockets’ druggability is a critical aspect to avoid intractable targets and to focus drug discovery efforts on sites offering better prospects. Since, in most cases, the same template was used for homology modelling, all the predicted structures were similar for each group of homologue proteins (see Supplementary Figure S2). Although we found slight differences between the predicted pockets, all the proteins were predicted to host a druggable (0.5 < DS ≤ 0.7) or highly druggable pocket (DS > 0.7). A visual example of the detected pockets for CDK and AKT homologues in *L. braziliensis* is presented in Figure 1. A summary of the best model per protein, including the template used, the QMEAN values and the calculated DS for the kinases of *L. braziliensis*, is available in Table 2. The rest of the variables and templates for the other species are available in Supplementary Tables S2–S8.

Another relevant feature to prioritize targets is determining whether or not the druggable pockets match the catalytic sites. In this sense, we assume that if the catalytic site overlaps with the druggable pocket where a putative drug would bind, it is more likely that the molecule can modulate the enzyme’s activity. The catalytic sites of each protein were predicted using InterPro [26]. For all the analysed proteins, except mTOR, at least one residue of the druggable pocket overlaps with residues of the respective catalytic site. A graphical example for AKT and CDK of *L. braziliensis* is shown in Supplementary Figure S3.

### 2.3. Hits Found by Molecular Docking

**Figure 2:**
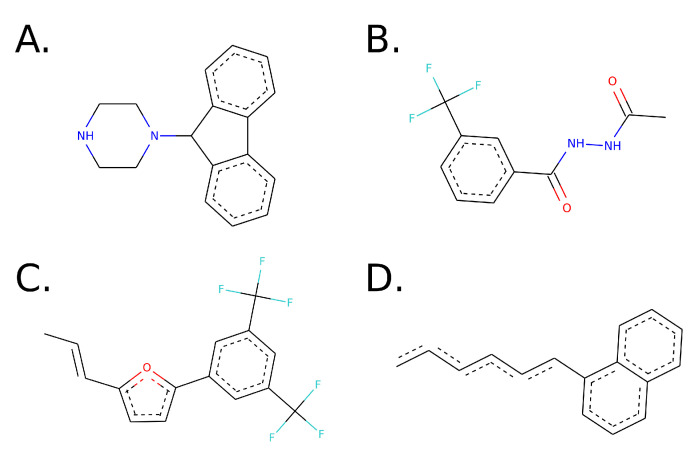
Maximum common substructures of the compounds detected during the virtual screening, split into cluster 1 with 15 molecules (**A**), cluster 2 with 8 (**B**), cluster 3 with 5 (**C**) and cluster 4 with 4 (**D**).

Using the best models per kinase in each *Leishmania* species, we subjected them to virtual screening using molecular docking, selecting as the binding site the most druggable predicted pocket. The complete scores and compound ID per model are available in Supplementary Table S9. To identify common substructure patterns, we clustered the best 20 compounds per model using chemical representations of the ligands. The identified substructures are shown in Figure 2.

A subset of the compounds was active against the majority of kinases, given the conservancy of the enzyme’s catalytic site. For these cases, we find challenges to identify candidates selective for these *Leishmania* proteins with regard to the human counterparts. However, the main priority is to find active molecules that can be later subjected to the control of potential toxicities using formulation strategies, among other methodologies [27]. In this sense, it is also important to check if any of the identified compounds share similar substructures with compounds tested as kinase inhibitors or with compounds similar to them by structural and physicochemical properties. Using the tool ligQ, we obtained a list of 2609 compounds, which was compared to the set prioritized by molecular docking using their molecular fingerprints. The full list of compound IDs and SMILES representations of their chemical structures is available in Supplementary Table S10. From this comparison, we detected an inhibitor of GSK3 (ZINC00027361) and two hits with similarities greater than 50% to molecules reported in the ChEMBL database [28]. Their corresponding IDs are shown in Table 3.

Regarding ZINC00027361 (i.e., known as TDZD-8), it has been studied as a human GSK3 beta inhibitor for different diseases [29]. The molecule is a selective non-ATP competitive inhibitor with a neuroprotective role, which has been evaluated in vivo through safe and nontoxic concentrations [30]. Finding this known human GSK3 inhibitor in our pipeline motivates the run of experiments that can hint not only at the novel activity of TDZD-8 towards *Leishmania* but also support, in an indirect way, the potential presence of one of the kinases (GSK3) in the parasite. Previous works following a similar strategy with the AKT-like kinase from *Leishmania* and the human AKT inhibitor X have been reported [31].

In the case of the other compounds shown in Table 3, ZINC19835187 was found in the top 20 for 80% of all the kinase models subjected to the screening, becoming a relevant candidate to further explore for drug discovery purposes. For the other selected molecules, some of them are novel scaffolds, and some others contain substructures previously assayed towards these enzymes. To check the latter, we selected a set of consensus hits towards GSK3 and ran two pipelines to predict potential molecular targets. Specifically, we used the SEA [32] and SwissTargetPrediction servers [33]. Based on the predictions, some of the molecules can be active toward GSK3 and other kinases, supporting their posterior study as kinase inhibitors.

Future work will include the validation of their potential inhibitory activity through in vitro/in vivo methodologies. For this manuscript, we have performed experimental analysis for the GSK3 inhibitor found during the virtual screening in order to understand the mechanism of death caused by this molecule in *Leishmania*.

### 2.4. Induction of Cell Death by GSK3 Inhibitor

**Figure 3:**
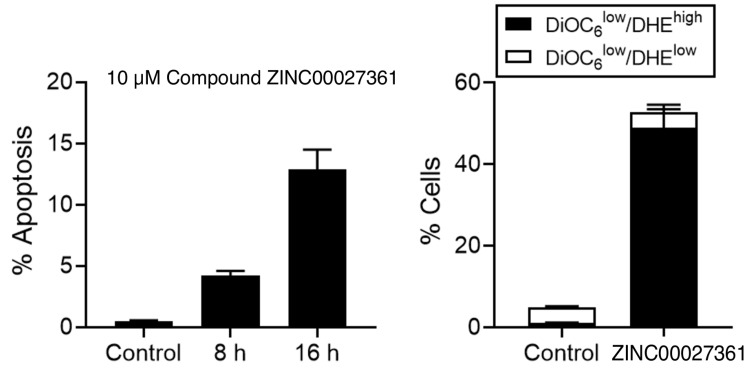
Time course of the GSK3 inhibitor (ZINC00027361) effect on parasites’ death. In the left side it is shown the cell cycle distribution of *L. panamensis* promastigotes based on the apoptosis percentage. Parasites were treated with 10 μM of ZINC00027361 for the times indicated, stained with propidium iodide, and the proportion of parasites in each phase of the cell cycle was quantified by flow cytometry as described in Methods. Parasites in the Sub-G1 region (hypodiploidy) represent apoptotic-like parasites. Untreated control parasites were run in parallel. In the right side, *L. panamensis* promastigotes were untreated (control) or treated with 10 μM inhibitor at 16 h, and parasites with disrupted $\Delta \Psi_{m}$ (DiOC6(3) low and ROS production (DHE)) were measured by flow cytometry. Data are shown as means of three independent experiments with their standard deviations.

First, we analysed the ability of ZINC00027361 in promoting apoptosis-like cell death in *L. panamensis* promastigotes, as assessed by DNA breakdown determined by flow cytometry. *L. panamensis* promastigotes were incubated with 10 μM of the compound for 8 and 16 h, and analysed by flow cytometry. Our results showed that the inhibitor did not have a significant effect on cell cycle after 8 h. However, parasites treated at 16 h underwent apoptosis, measured as hypodiploid parasites (Figure 3). The inhibitor induced DNA breakdown after 8 h incubation with *L. panamensis* promastigotes, and the percentage of parasites with hypodiploid DNA content (Sub-G0/G1 cell population) increased with the incubation time (Figure 3), suggesting an apoptosis-like cell death.

We next examined the effect of the inhibitor in mitochondrial-related processes in *L. panamensis* promastigotes. The ROS generation was monitored through the conversion of nonfluorescent dihydroethidine (HE) into red fluorescent ethidium (Eth) after its oxidation via ROS and changes in $\Delta \Psi_{m}$ through the accumulation of the fluorescent cationic probe DiOC6(3) (green fluorescence), which depends on the mitochondrial potential. As shown in Figure 3, untreated parasites exhibited a high $\Delta \Psi_{m}$ (DiOC6(3)high), and the levels of intracellular ROS were low. The inhibitor induced an increase in the percentage of cells and loss in $\Delta \Psi_{m}$. Taken together, our data suggest a role of mitochondria in the anti-*Leishmania* activity of ZINC00027361.

Studying ZINC00027361 in *Leishmania* promastigotes is useful because if a chemical inhibitor has an effect on them, this inhibitor could be used as a prophylactic compound for people who do not live in an endemic area. We expect that in similar studies in amastigotes, a good correlation will be observed as has been reported so far in our studies with other kinases, such as AKT-like, where the effect on promastigotes correlates with the effects on amastigotes in vitro [23,31]. The kinases, in general, are well conserved in the different cellular stages due to their roles for molecular signalling. Therefore, they are well represented in the different stages of the parasite throughout its biological cycle. It is known that trypanosomatids carry out a global transcription of most of their messenger RNAs (mRNA), evidencing an apparent lack of transcriptional regulation and a preference for post-transcriptional and translational regulatory processes [34], which reinforces the idea that the regulatory system based on kinases could have a fundamental role in the control and regulation of transcription and translation processes and protein stability, among others. Our findings, and the knowledge that approximately 2% of the complete genome of these parasites codes for kinase-type proteins [35], reinforce the idea that phosphorylation mechanisms are part of fundamental processes in the biology of these organisms at different stages.

In the experiment, we see the inactivation of the signalling pathway, causing the death of the parasite. This indicates that when inhibiting the kinase, there is a type of apoptosis-like death (damage to genetic material) and loss of cell membrane potential, which leads to damage to the parasite mitochondria. These experiments are sufficient to additionally characterize associated death with kinase inhibition in at least one of the selected kinases.

### 2.5. Protein Interactions Associated with the Selected Kinases

**Figure 4:**
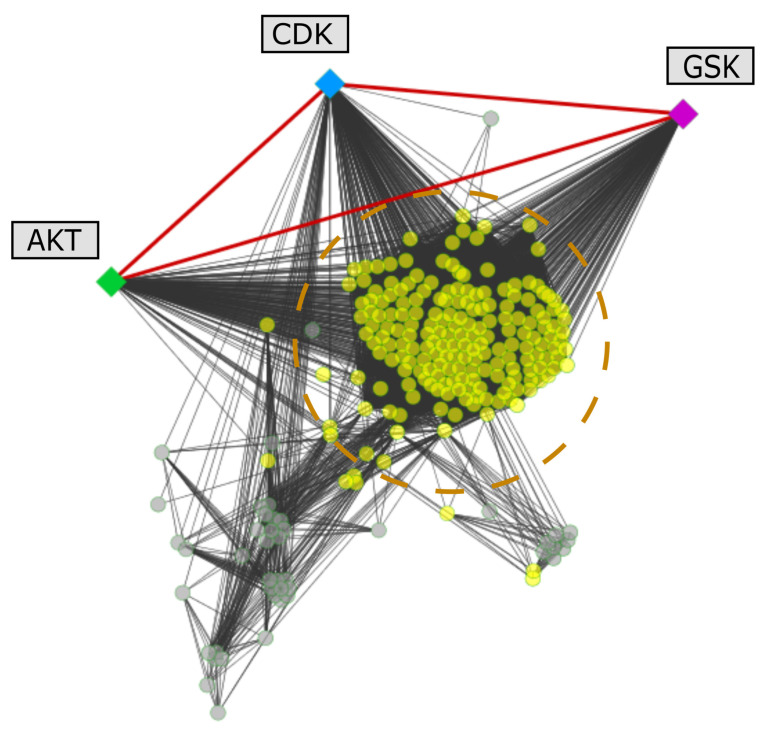
Visualization of the parasite-predicted PI3K-AKT pathway proteins, including the set of kinases, in the protein–protein interaction network of *L. major*. The kinases within the whole network are represented as cyan nodes, and Hsp90 members as red nodes.

The selected kinases prompted us to analyse additional information about the pathways they are involved in and how essential these might be for the biology of the parasite. Some *Leishmania* kinases are directly involved in these routes and are highly interconnected according to interactions detected in previously built protein–protein interaction (PPI) networks of three of the included species (*L. major*, *L. infantum* and *L. braziliensis*). As an example, we mapped the kinases and other detected proteins of the PI3K/AKT pathway onto the PPI network of *L. major* (Figure 4).

**Figure 5:**
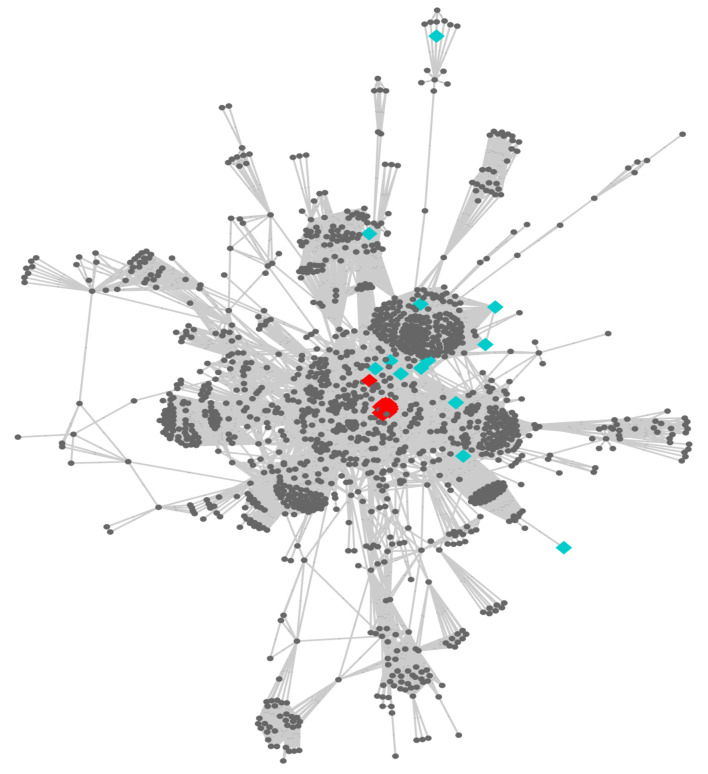
Subnetwork of interactions associated with the triad of identified kinases GSK (magenta), AKT-like (green) and CDK (blue) from *L. major*. The three proteins are directly interconnected and regulate relevant pathways on the parasite (yellow nodes) that are potentially associated with the PI3K/AKT pathway.

According to Figure 4, a group of the proteins, including the kinases, are in the centre of the network, where the node’s degree values are higher compared to the rest. In addition, 10 proteins, including AKT, CDK and GSK3, are directly connected with each other in the network. We illustrate in Figure 5 a subnetwork that explains the direct associations among these kinases and the synchronized regulation of a large cluster of proteins that, according to our bioinformatics analysis, are involved in the PI3K/AKT pathway.

We mapped the clusters of the proteins detected in the pathways with the reconstructed protein interaction networks of *L. major*, *L. infantum* and *L. braziliensis* (see Supplementary Figure S1). The proteins detected were NF-κβ, c-myb, Cyclin, CDK, JAK, AMPK, S6K1, PTEN, PHLPP, PI3K, AKT, GSK3, ATM, eIF4E, Hsp90, mTOR and 14-3-3, which are currently accepted targets for anticancer research therapy. Details about the detected proteins are available in the Supplementary Text. These proteins, when targeted specifically, can induce cell apoptosis in higher eukaryotes. All these proteins constitute potential molecular targets for leishmaniasis chemotherapy.

The protein networks allowed us to identify that the cell cycle (PI3K/AKT) and potential apoptotic protein interactions of *Leishmania* share, in general, similarities with those in mammalian cells, including kinases. Although targeting these PI3K pathway nodes individually is appealing, unexpected positive feedback loops [36] or the activation of compensatory signalling [37] can lead to apoptosis evasion. Therefore, a regimen such as dual or combinatorial therapy that targets either different (parallel targeting) or the same signalling pathways at several key nodes (vertical targeting) should be considered. This approach may also prevent the appearance of drug resistance.

Finally, in order to rank the detected kinases, we calculated topological metrics from the protein networks, such as the degree and the capability of being a bottleneck within the network. These metrics have been associated with the potential essentiality of the protein [38]. An example of the values calculated for the kinases of *L. braziliensis* is in Table 4.

We found four of the five kinases in the annotated network. All the protein kinases are highly interconnected, and two of them, CDK and AKT, are classified as key bottlenecks within the network. Another relevant bottleneck is PI3K (not shown), which, despite not being highly connected, is relevant in the cross-talk between the potential PI3K/AKT and potential apoptosis pathways in the parasite.

To facilitate the reproduction of the computational pipeline or run additional analysis, a GitHub repository (https://github.com/rochoa85/data-Biomolecules-Kinases accessed on 7 July 2021) is available with data of the models, the protein networks and scripts to run the comparative analysis and clustering of the selected compounds.

### 2.6. Literature Search of the Selected Kinases

One relevant protein kinase found is AKT, a pivotal therapeutic target in cancer research [39,40], which is a serine/threonine kinase that mediates cell survival by inducing cell proliferation and blocking apoptosis. AKT blocks apoptosis by inhibiting caspase 9 and thus apoptosome complex formation, a key initial step of the intrinsic apoptotic pathway [41], or by phosphorylating other proteins such as the proapoptotic regulator BAD, which binds to 14-3-3 and, in turn, releases the antiapoptotic proteins Bcl2 and Bcl-X [42]. On the other hand, AKT promotes cell survival by activating the stress protein kinase pathways (SAPK), MAPK kinase pathway and JNK pathway [43], enhancing the Mdm2-mediated ubiquitination and degradation of p53 [44], or by phosphorylating transcriptional factors such as FoxOs or NF-κβ [45] that regulate the expression of proapoptotic members of the Bcl-2 family [46]. Some mechanisms can potentially be translated to its trypanosomatid counterpart, which can activate, as tumour cells do, survival pathways under drug-induced or stress conditions or conditions defined by transient lack of nutrients, limited oxygen supply and changes in pH. Survival to these changes is essential for cell cycle and disease progression in human and nonhuman reservoirs. Specific drug inhibitors of either the mammalian PI3K/AKT pathway (i.e., perifosine or NVPBEZ235) or the *Leishmania* AKT (UBMC1-4) [23,24] have been proven to be effective against trypanosomatids, inducing apoptosis cell death and negatively affecting intracellular proliferation of amastigotes [13].

Cyclic-dependent kinase (CDK) orthologues were detected, which coordinate by phosphorylation of target protein checkpoints in cell cycle progression, apoptosis, differentiation and transcription [47]. Some CDK-related kinases (CRKs) and few cyclins have been characterized in *Leishmania* [48,49] and therefore constitute evidence for the evolutionary conservation of the basic cell cycle machinery. In addition, different research works have validated *Leishmania* CRK3 as a drug target [50,51].

The mechanistic target of rapamycin (mTOR) was also identified and has been proven to be essential in *L. major* [52]. mTOR is a downstream effector of PI3K/AKT that plays a key role in autophagy, cell cycle progression and proliferation. Inhibition of mTOR decreases phosphorylation of two downstream targets, 4E-BP1 and S6K, resulting in inhibition of protein synthesis [53], which does not necessarily induce apoptosis, but it can be considered in combination with other drugs as it induces cell arrest and sensitizes cells to apoptosis [54]. mTOR and PI3K inhibitors that are currently in preclinical and clinical development showed anti-*Leishmania* activity by altering cell size and inducing cell cycle arrest [13]. Human dual PI3K and mTOR inhibitors (i.e., NVP-BEZ235, PI-103) have been shown to enhance chemotherapeutic effects in cancer cells and higher activity in *Leishmania* than mTOR inhibitors alone [55]. There is a collection of dual inhibitors, either of PI3K-mTOR or mTOR-AKT, in various phases of clinical development [56], worthy of consideration in anti-*Leishmania* therapy.

AMP-activated protein kinase (AMPK) was another identified kinase, which is a major regulator of energy metabolism activated in response to energetic stress (any stimuli increasing the cellular AMP/ATP ratio) [57], thereby inhibiting all anabolic pathways involved in cell growth. AMPK can exert either a pro- or antiapoptotic effect. For example, as an inductor, AMPK activation may result in p38 MAPK-mediated translocation of the proapoptotic Bax into the mitochondria, inhibition of cell cycle progression by upregulating p53 [58] or inhibition of mTOR. Several AMPK activators exhibit antiproliferative activity in different cancer cell lines, and at least one, Metformin, is currently used in the clinic to treat diabetes and polycystic ovary syndrome [59].

Finally, GSK3 is a downstream substrate of PI3K/AKT and a ubiquitous serine/threonine kinase highly conserved among eukaryotes. This enzyme is a key regulator of several cellular processes, including cell cycle regulation, glucose metabolism or apoptosis, and is currently under intense investigation. Moreover, depending on the signalling pathway involved, GSK3 has the capability of modulating the apoptotic/survival threshold of the cell, thus having a two-fold role in apoptosis, either promoting the mitochondrial-mediated intrinsic or inhibiting the death receptor-mediated extrinsic signalling pathways [60]. Despite the contradictory role of GSK3 as a tumour suppressor or promoter, depending on the type of cancer [61], it is thought that GSK3 inhibitors will eventually be used to suppress the proliferation of certain cancers [62]. The enzyme has been shown to be essential for cell viability and is differently expressed and located in each parasite stage, thus playing various roles in response to stress conditions [63]. However, its specific molecular context has not yet been elucidated. Some studies point out molecular differences between human and *Leishmania* GSK, but its catalytic domain is highly conserved in eukaryotes despite some slight not crucial differences in the ATP binding pockets [64], which makes it possible to consider drug repurposing and detection of novel hits through virtual screening approaches.

## 3. Materials and Methods

### 3.1. Selection and Comparison of Human and Leishmania Kinase Sequences

Kinases and other proteins from the PI3K/AKT (KEGG:04151) pathway reported in humans were identified from the KEGG database [65]. The sequences were downloaded from UniProtKB [66]. Then, we mapped potential orthologues in *Leishmania* species using the complete set of annotated proteins from *L. major* (cutaneous), *L. infantum* (visceral and cutaneous), *L. braziliensis* (cutaneous and mucocutaneous), *L. donovani* (visceral) and *L. mexicana* (cutaneous), available in the TriTrypDB database (version 4.0) [67]. With the data collected, only annotated kinases based on in silico protocols were included, leaving out nonfunctional proteins or pseudogenes.

To compare the proteins at sequence level, a best reciprocal hits (BRH) approach based on the BLAST algorithm was used [68]. The criteria to select potential orthologues were identity percentages > 40% and E-values < 1 × 10${}^{-10}$ using as query *Leishmania* or human protein sequences. After that, a list of *Leishmania* kinases likely involved in the signalling pathway of interest was proposed.

### 3.2. Prediction of Ligandibility Metrics for the Leishmania Kinases

To prioritize the parasite kinases used in the screening, a ligandibility prediction model was built using bioactivity data of annotated kinases available in the ChEMBL database [28]. Ligandibility is a novel concept of effort and reward in drug discovery, where a target is highly ligandable if little effort is required to generate a high-affinity inhibitor [69]. The value can be calculated per target based on available $K_{i}$ values and the total number of ligands, defined as

(1) $$\mathrm{Lig}=\frac{pK_{i}>7}{N},$$

where the upper part represents the number of ligands reporting $pK_{i}$ values greater than a threshold of 7, and the lower part *N* is the total number of assayed ligands. The threshold of 7 was determined because it is the value able to maximize the variance and thus provide the best discrimination between targets [69]. To be robust, a minimum of 100 $K_{i}$ data points per target are required to calculate the metric. The calculated values of the reported kinases were associated with those of the parasite homologues, and those with values higher than 0.5 were prioritized for subsequent screening.

### 3.3. Generation of Structural Homology-Based Models and Druggability Assessment

Given that most of the identified kinases do not report protein structures at the Protein Data Bank (PDB) [70], we attempted to build homology-based models using our own structural genomic pipeline [71]. Up to three PDB structures were used by SWISS-MODEL software [72] as templates for homology-based modelling, and five models per template for each protein were constructed. One representative model was chosen based on the maximization of the QMEAN Z-score function [73], with a range between −4 and 4.

Druggability score (DS) describes the ability of a given protein to bind a drug-like compound [74]. Druggable proteins should have a well-defined pocket with suitable physicochemical features to allow drug binding site prediction. Structural druggability of each potential target was assessed by using the fpocket program [75]. Based on a preliminary analysis of DS distribution for all pockets that host a drug-like compound in the PDB [76,77], pockets are classified into four categories: (i) non druggable (ND; DS ≤ 0.2), (ii) poorly druggable (PD; 0.2 < DS ≤ 0.5), (iii) druggable (D; 0.5 < DS ≤ 0.7) and (iv) highly druggable (HD; DS > 0.7). This information was used to prioritize them as potential molecular targets in different species of the parasite. Finally, the centroid of the pocket was obtained for preparing the molecular docking analysis.

### 3.4. Molecular Docking Screening

The structures of the different kinases across the five *Leishmania* species were subjected to a virtual screening campaign with approximately 46,000 drug-like compounds and reported inhibitors selected from the ZINC database [78]. Before running the simulations, both protein and ligands were parametrized using AutoDock Tools through the addition of hydrogen bonds to polar side chains and the estimation of partial charges using the Gasteiger methodology [79]. The simulations were run with AutoDock Vina [80] using the DrugDiscovery@TACC portal to automatize the calculations (http://drugdiscovery.tacc.utexas.edu accessed on 12 March 2021). The results were filtered based on the higher negative scores for candidate ligands. In addition, SMILES representations of the best 20 compounds per target per species were used as input to cluster the chemical structures using the Taylor-Butina clustering [81] with Morgan fingerprint representations, available on the RDKit (www.rdkit.org accessed on 11 April 2021). A 80% similarity threshold was selected based on the Tanimoto index [82]. For each predicted cluster, a chemical representation of the compounds was obtained using the maximum common substructure method also available on the RDKit [83]. The results were compared with ligands from bioactivity databases that are similar to inhibitors bound to kinase crystal structures reported in the PDB, using the ligQ tool [84]. One of the ligands (ZINC00027361), reported as a GSK3 inhibitor, was detected by our method. Because of this, the potential anti-*Leishmania* activity was validated experimentally.

### 3.5. Analysis of Apoptosis-Like Cell Death by Flow Cytometry Using the GSK3 Inhibitor

The Leishmania strains used in this study were *L. panamensis* (MHOM/CO/87/UA140), kindly provided by Dr. Iván Vélez from the Programa de Estudio y Control de Enfermedades Tropicales, PECET. Based on the latest *L. panamensis* genome projects, it shares the kinase homologues found in the other species [85]. *Leishmania* promastigotes were grown at 26 °C in RPMI-1640 culture medium (Invitrogen, Carlsbad, CA), supplemented with 10% foetal bovine serum (FBS), 2 mM L-glutamine, 100 U/mL penicillin and 100 μM streptomycin. Promastigotes were treated at 26 °C with the indicated compound during their logarithmic growth phase (1.5 × $10^{6}$ parasites/mL). Late stationary promastigotes were obtained after incubation of the parasites for more than 6 days with a starting inoculum of 1 × $10^{6}$ parasites/mL. The *L. panamensis* promastigotes were incubated in the absence or presence of the indicated concentrations of ZINC00027361 for different incubation times and then analysed for DNA breakdown by flow cytometry, using a fluorescence-activated cell sorting (FACS) Calibur flow cytometer (Becton Dickin-son, San Jose, CA, USA), as previously described [86]. Quantification of apoptotic-like cells was monitored following cell cycle analysis as the percentage of cells in the sub-G0/G1 region, representing hypodiploids or apoptotic-like cells [31].

A cytofluorimetric analysis of mitochondrial transmembrane potential ($\Delta \Psi_{m}$) and generation of reactive of oxygen species (ROS) was conducted. A total of 2 × $10^{6}$ Leishmania parasites were pelleted by centrifugation, washed with PBS and incubated in 1 mL PBS containing 20 nM 3,30-dihexyloxacarbocyanine-iodide (DiOC6(3), green fluorescence; Molecular Probes, Leiden, The Netherlands) and 2 μM dihydroethidine (HE, red fluorescence after oxidation; Sigma) at room temperature and darkness for 20 min, and then analysed on a Becton Dickinson FACSCalibur flow cytometer as previously described [86].

### 3.6. Mapping of Selected *Leishmania* spp. Proteins on Protein-Protein Interaction Networks

To finally understand the role and essentiality of the selected *Leishmania* kinases, these were mapped into protein–protein interaction networks previously built in house [25]. For each protein, different neighbours were identified to annotate the signalling pathway. After the mapping, topological properties, such as betweenness, centrality and degree levels, were calculated using the plugin Network Analyzer (release 2.7) from Cytoscape [87].

Then, based on the literature evidence about the presence of relevant signalling pathways in *Leishmania* spp., a controlled search was performed to identify if some of the selected kinases, and other proteins they interact with, could be part of these routes on the parasite. For this purpose, the PubMed search engine was used with the terms cancer, therapy, apoptosis, cell death, survival, PI3K, AKT and *Leishmania*. The extracted data were exhaustively reviewed and associated with the bioinformatics findings.

## 4. Conclusions

Cell cycle, survival and potential apoptosis are tightly interconnected conserved signalling pathways essential for the biology of *Leishmania*. It is generally accepted that kinetoplastids have evolved signalling pathways highly divergent from those of metazoans, and the studied cell processes in *Leishmania* share kinase homologues with these signalling pathways, such as the human PI3K/AKT molecular routes. Despite the efforts to characterize these processes, detailed information about the molecular pathways and proteins involved in *Leishmania* remains largely unknown. However, some key components report a considerable number of clues that allow it to be inferred that, for example, the chosen kinases are expressed by the parasite.

The bioinformatics findings can be used to facilitate the study of these enzymes and the discovery of novel chemical entities and for drug repurposing in anti-*Leishmania* therapy, since there is a promising list of specific drugs already licensed for human use. Overall, our data support the existence of *Leishmania* cell cycle proteins that potentially contribute, with a deep characterization of the major kinases involved in the pathways. Therefore, the knowledge of well-detailed signalling networks will potentially support drug development, repositioning and the design of combinations or multitarget drugs (MTD) to fight the parasite, thus preventing resistance and/or driving treatments to patients sooner, safer and cheaper.

## Figures and Tables

**Table 1:** List of kinases present in the human PI3K/AKT pathway with potential orthologues (UniProtKB IDs) in different *Leishmania* species.

Human Gene KEGG	*L. mexicana* ID	*L. major* ID	*L. braziliensis* ID	*L. infantum* ID	*L. donovani* ID
AKT	E9ARP5	Q27687	A4H9L8	A4HXY2	E9BDT9
GSK3	E9ARG4	Q4QE15	A4H9D1	A4HXQ3	E9BDK8
AMPK	E9ALM1	E9AE64	A4HHK1	A4I4Q9	E9BL11
mTOR	E9AU66	Q4Q0C8	A4HBF9	A4IE36	E9BV14
CDK	E9ASH4	O96526	A4HNR5	A4ICT0	E9BTB9

**Table 2:** Summary of the results from the modelling (QMEAN), ligandibility (Lig) and druggability (DS) calculations for the selected kinases of *L. braziliensis*.

Kinase	PDB Template	Lig	QMEAN	DS
CDK	6GU6.A	0.654	−1.71	0.727
AKT	4WB5.A	0.741	−0.44	0.509
mTOR	4JSN.A	0.791	−3.76	0.533
AMPK	5EZV.A	0.524	−1.07	0.571
GSK3	3E3P.A	0.516	0.56	0.625

**Table 3:** Compounds detected by molecular docking in all the kinase models. The compounds share substructures with ligands found in the list of molecules similar to known kinase inhibitors.

ZINC ID—Docking Results	ChEMBL ID—ligQ Results	Similarity
ZINC00027361	CHEMBL284861	1.000
ZINC00135232	CHEMBL1989856	0.583
ZINC00135232	CHEMBL2000433	0.625
ZINC19835187	CHEMBL1569442	0.515

**Table 4:** Summary of topological metrics for the selected kinases in the protein interaction network of *L. braziliensis*.

Kinase	Degree	Bottleneck
CDK	159	45676
AKT	140	29858
mTOR	–	–
AMPK	170	41
GSK3	132	31

## Data Availability

Data of the models, the protein networks and scripts to run the comparative analysis and clustering of the selected compounds is available at: https://github.com/rochoa85/data-Biomolecules-Kinases (accessed on 7 July 2021).

## Supplementary Materials

The following are available online at https://www.mdpi.com/article/10.3390/biom11071037/s1, Figure S1: Protein-protein interaction networks of *Leishmania major*, *Leishmania braziliensis* and *Leishmania infantum*. Figure S2: Protein structure visualization for CRK in different Leishmania strains. Figure S3: Superposition of the reported catalytic site and the predicted pocket for CDK and AKT homologues in *Leishmania braziliensis*. Table S1: List of all the proteins present in the human PI3K/AKT and apoptosis pathways with potential orthologues in different *Leishmania* species. Tables S2–S8: Summary of the modelling variables and the druggability score for the kinases detected in the five species of *Leishmania* analysed. Table S9: List of best 20 compounds per kinase model based on the AutoDock Vina score. Table S10: Full list of compounds IDs and SMILES representations of their chemical structures.
