# Supplementary material for: Identification of Potential Kinase Inhibitors within the PI3K/AKT Pathway of Leishmania Species
Source: Biomolecules. 2021 Jul 16;11(7):1037. doi: 10.3390/biom11071037 (PMC8301987; doi:10.3390/biom11071037)
Supplement: Supplementary file 1 [file biomolecules-11-01037-s001.zip › supplementary_information_Ochoa_et_al_2021.pdf]

Supplementary Information

Identification of potential kinase inhibitors within the PI3K/AKT pathway of *Leishmania* species

Rodrigo Ochoa[1,2], Amaya Ortega-Pajares[3], Florencia A. Castello[4], Federico Serral[4], Darío Fernández do Porto[4,5], Janny A. Villa-Pulgarin[6], Rubén E. Varela-M[7], Carlos Muskus[1]

[1]Programa de Estudio y Control de Enfermedades Tropicales, PECET, Universidad de Antioquia, Medellín 050010, Colombia

[2]Biophysics of Tropical Diseases, Max Planck Tandem Group, University of Antioquia, Medellín 050010, Colombia.

[3]Department of Medicine and Radiology, Peter Doherty Institute, University of Melbourne, Melbourne, VIC, Australia

[4]IC-CONICET, Ciudad Universitaria, Pabellón 2, C1428EHA Ciudad de Buenos Aires, Argentina

[5]Departamento de Química Biológica, Facultad de Ciencias Exactas y Naturales, Universidad de Buenos Aires, Ciudad Universitaria, Pabellón 2, C1428EHA Ciudad de Buenos Aires, Argentina

[6]Grupo de Investigaciones Biomédicas, Facultad de Ciencias de la Salud, Corporación Universitaria Remington, Medellín 050034, Colombia

[7]Grupo de Investigación en Química y Biotecnología (QUIBIO), Facultad de Ciencias Básicas, Universidad Santiago de Cali, Cali 760035, Colombia

Supplementary Figures

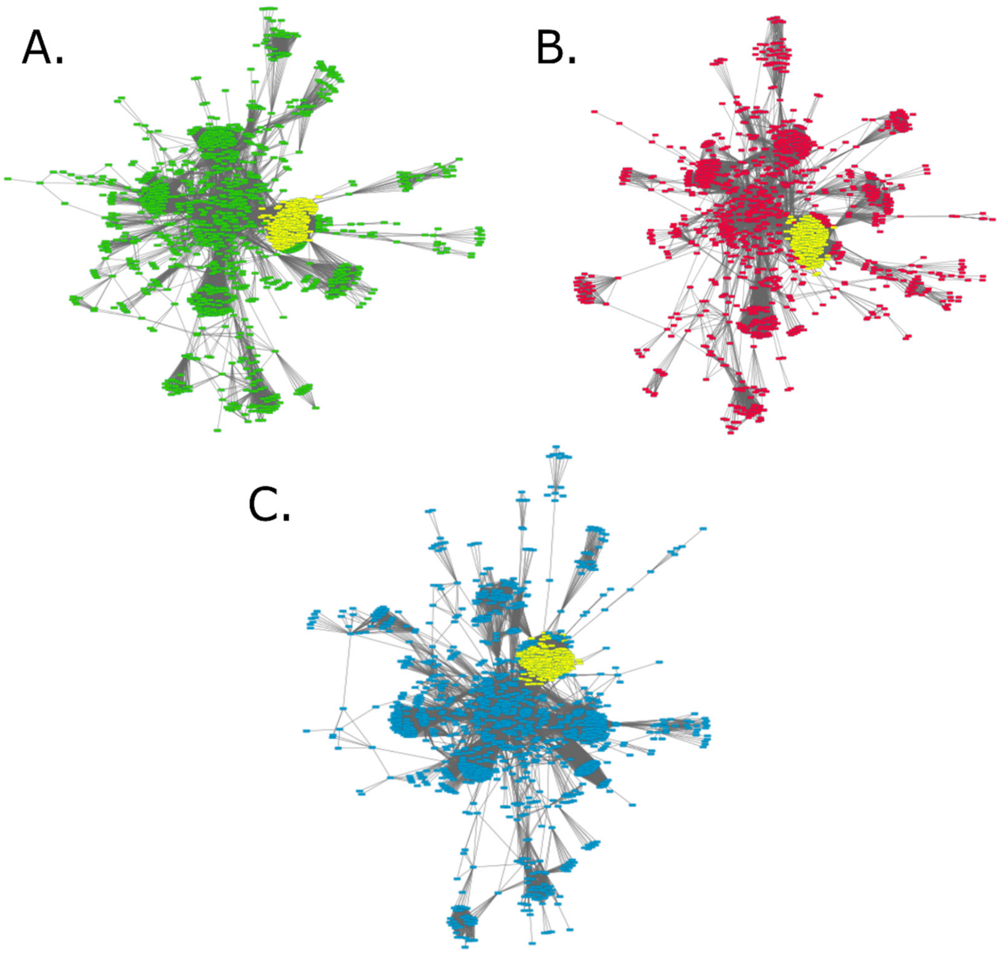

Figure S1. Protein-protein interaction networks of (A) *Leishmania major*, (B) *Leishmania braziliensis* and (C) *Leishmania infantum*. The yellow nodes represent the sub-network of proteins with interactions to GSK3, which explain the high connectivity degree the protein has within *Leishmania* spp. pathways.

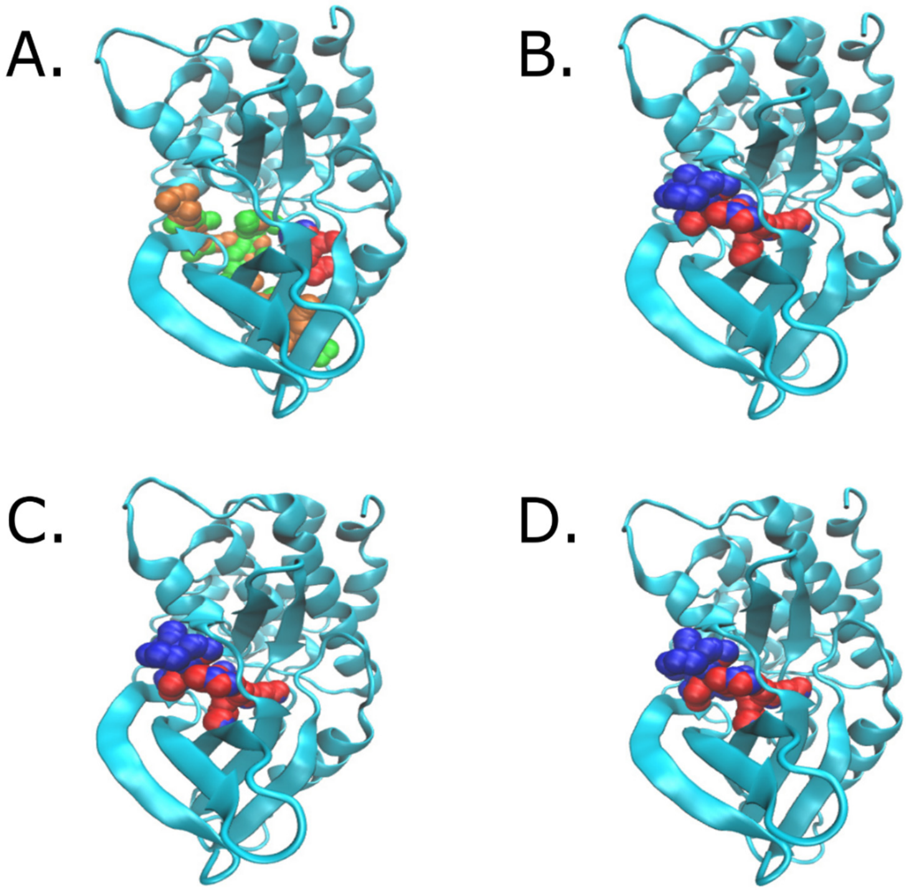

Figure S2. Protein structure visualization for CRK in different *Leishmania* strains. (A) *L. major*, (B) *L. mexicana*, (C), *L. infantum*, (D) *L donovani*. The most druggable pocket is shown. Polar alpha spheres are depicted in red while apolar alpha spheres in blue. Predicted most druggable pockets were equivalent for all the strains analysed except the pocket predicted for the *L. major* CRK structure where the folding forms a new a pocket with higher druggability score. In this case, the equivalent pocket to the most druggable pocket of the other proteins is also shown in green (apolar alpha-spheres) and yellow (polar alpha-spheres).

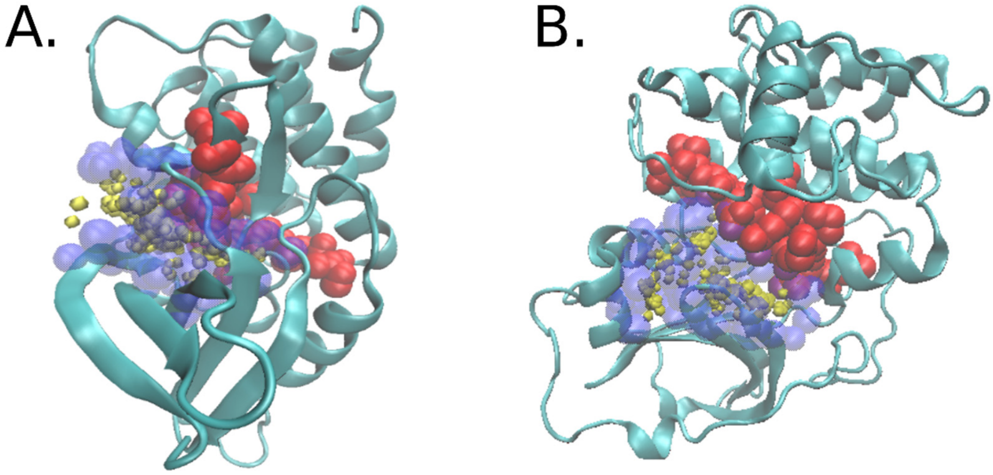

Figure S3. Superposition of the reported catalytic site and the predicted pocket for CDK (A) and AKT (B) homologues in *L. braziliensis*. Catalytic residues are depicted in red, pocket residues in blue, and the C-α atoms of the pocket in yellow.

Supplementary Tables

Table S1. List of all the proteins present in the human PI3K/AKT and apoptosis pathways with potential orthologues (UniProtKB IDs) in different *Leishmania* species.

	Cutaneous		Cutaneous/ Mucocutaneous	Cutaneous/ Visceral	Visceral
**Human gene KEGG***	***L. mexicana* ID**	***L. major* ID**	***L. braziliensis* ID**	***L. infantum* ID**	***L. donovani* ID**
PHLPP	E9AZD9	E9ACV0	A4HG10	A4I329	E9BJF5
Rac1	E9ARM6	Q4QGD2	A4HLP5	E9AGQ4	E9BDR9
MEK	E9ASK6	Q4Q1Z0	A4HNU6	A4ICP8	E9BTF2
Rheb	E9B2Y3	Q4Q573	A4HKJ3	A4I825	E9BNW8
Raptor	E9AXD8	Q4QA34	A4HEF7	A4I1A2	E9BHG6
AKT	E9ARP5	Q27687	A4H9L8	A4HXY2	E9BDT9
GSK3	E9ARG4	Q4QE15	A4H9D1	A4HXQ3	E9BDK8
AMPK	E9ALM1	E9AE64	A4HHK1	A4I4Q9	E9BL11
PTEN	E9B4W8	Q4Q359	A4H7T1	A4I9V5	E9BQV4

14-3-3	E9ANI1	Q4QH45	A4H6F2	A4HUU6	E9BAM3
mTOR	E9AU66	Q4Q0C8	A4HBF9	A4IE36	E9BV14
eNOS	E9AZU3	Q4Q8E2	A4HGH1	A4I3K3	E9BJK6
PP2A	E9AUK9	Q4QCX5	E9AIC2	A4HYP6	E9BEL9
Cyclin	E9B3B5	Q4Q4U0	A4HE79	A4I8F7	E9BPA2
CDK	E9ASH4	O96526	A4HNR5	A4ICT0	E9BTB9
Ras	E9AYX8	E9AD53	A4HFK0	E9AHE8	E9BIZ5
Hsp90	E9B3L2	Q4Q4I6	A4HL70	E9AHM8	E9BPW4
JAK	E9AUL5	Q4QCW9	--	A4HYP1	E9BEM5
S6K1	E9AVH4	E9AF23	A4HLC0	A4HZI4	--
Gby	E9AN88	Q4QHD6	--	A4HUJ0	E9BAC8
NF-κB	--	Q4QAW7	A4HD71	E9AH25	E9BGM5
ERK	--	Q4Q449	A4HLJ9	A4I910	E9BQ78
PI3K-Class IB	E9APJ6	E9AE04	A4HDR3	--	--
PI3K-Class IA	--	--	--	A4HVU4	E9BBN7
SGK	E9B6S9	Q9GNR4	A4HN71	--	--
eIF4E	--	Q4QD60	A4HA98	A4HYH8	--
ECM	--	--	A4HBI8	E9AHT7	E9BPZ2
c-myb	--	--	A4H921	--	
ACTG1	E9AKA4	Q9U1E8	A4H438	A4HSC2	E9B890
NFKBIA	E9B1A1	Q4QAW7	A4HD71	A4I610	E9BM74
CYC	E9AQU2	Q4QEN5	A4H8R0	A4HX29	E9BCZ1
MAP2K1	E9ASK6	Q4Q1Z0	A4HNU6	A4ICP8	E9BTF2
AKT3	E9ARP5	Q27687	A4H9L8	A4HXY2	E9BDT9
EIF2S1	E9AJY2	E9ACP3	A4H3R0	A4HRY4	E9B7W6
TUBA1A	E9AP62	Q4QGC5	A4H727	E9AGK7	E9BBA8
MAP3K5	E9B179	Q4Q6Y5	A4HIN9	A4I5Y6	E9BM52
CAPN1	E9B1J6	Q4Q6L7	A4HJ24	A4I6E6	E9BMH1
ATM	E9AJH9	E9AC86	A4H3A6	A4HRL5	E9B7F7
CTSB	E9ALZ5	E9ADT5	A4HH90	A4I4D6	E9BKN2
ENDOG	E9AN71	Q4QHF4	A4H653	A4HUH3	E9BAB1
CTSF	E9AMH7	Q4QI68	A4H5E5	A4H5E6	Q7K8Z6
PTPN13	E9ATX2	Q4Q0M4	A4HQ52	A4IDV1	E9BUR8
EIF2AK3	E9B501	Q4Q2Y5	A4HAS7	A4I9Y7	E9BQZ0
ACTB	E9AKA4	Q9U1E8	A4H438	A4HSC2	E9B890
IKKA	E9AV60	--	--	--	--
IKKB	E9AUN4	Q4QCV0	--	A4HRW6	E9BEP4

APAF	E9AX72	--	--	--	--
XIAP	E9ATG5	Q4QBN7	A4HPQ0	A4ID13	E9BUB0

For the following tables (S2 to S8) these are the conventions used to name the columns:

GMQE: Accuracy of the tertiary structure of the model (from 0 to 1)

QMEAN: Metric to measure the quality of the model (> -4.0 are better models)

COV: Coverage of the alignment with the template

ID: Percentage of identity with the template

RES: Resolution of the crystal template

Ligands: Ligands co-crystallized in the template

fpocket: Pocket chosen by the fpocket tool to score the druggability

DS: Druggability score

Table S2. Summary of the modelling variables and the druggability score for the CDK kinases detected in the five species of *Leishmania* analysed.

UniProt ID	PDB template	GMQE	QMEAN	COV	ID	RES	Ligands	fpocket	DS
	6gu6.1.A	0,78	-1,52	0,93	55,9	2,33A	1QK	pocket 1	0,727
	6gu7.2.A	0,77	-2,38	0,93	55,9	2,75A	None	pocket 1	0,829
A4HNR5	1h1p.2.A	0,77	-1,88	0,94	59,45	2,10A	CMG	pocket 1	0,687
	6gu6.1.A	0,77	-1,53	0,93	54,86	2,33A	1QK	pocket 11	0,721
	6gu7.2.A	0,77	-2,41	0,93	54,86	2,75A	None	pocket 1	0,775
O96526	1h1p.2.A	0,77	-1,85	0,94	59,11	2,10A	CMG	pocket 1	0,664
	6gu6.1.A	0,77	-1,95	0,93	55,21	2,33A	1QK	pocket 1	0,678
	6gu7.2.A	0,77	-2,36	0,93	55,21	2,75A	None	pocket 1	0,775
	1h1p.2.A	0,77	-1,77	0,94	59,11	2,10A	CMG	pocket 1	0,687
E9ASH4	1h24.2.A	0,76	-2,13	0,94	59,11	2,50A	None	pocket 1	0,764
	6gu6.1.A	0,77	-1,53	0,93	55,21	2,33A	1QK	pocket 2	0,627
	6gu7.2.A	0,77	-2,36	0,93	55,21	2,75A	None	pocket 1	0,775
A4ICT0	1h1p.2.A	0,77	-1,76	0,94	59,11	2,10A	CMG	pocket 1	0,687
	6gu6.1.A	0,77	-1,53	0,93	55,21	2,33A	1QK	pocket 2	0,627
E9BTB9	1h1p.2.A	0,77	-1,76	0,94	59,11	2,10A	CMG	pocket 1	0,687

Table S3. Summary of the modelling variables and the druggability score for the AKT kinases detected in the five species of *Leishmania* analysed.

UniProt ID	PDB template	GMQE	QMEAN	COV	ID	RES	Ligands	fpocket	DS
	4wb5.1.A	0,77	-0,51	0,95	52,06	1,64A	ATP - MG	pocket 1	0,509
A4H9L8	6ccy.1.A	0,74	-2,11	0,97	40,81	2,18A	EX4	pocket 2	0,697
	4wb8.1.A	0,77	-0,45	0,95	51,75	1,55A	ATP - MG	pocket 1	0,769
Q27687	6ccy.1.A	0,74	-2,35	0,97	41,12	2,18A	EX4	pocket 2	0,704
	4wb8.1.A	0,77	-0,47	0,95	51,9	1,55A	ATP - MG	pocket 1	0,759
E9ARP5	6ccy.1.A	0,74	-2,31	0,98	39,51	2,18A	EX4	pocket 1	0,592
	4wb5.1.A	0,77	-0,51	0,95	51,43	1,64A	ATP - MG	pocket 2	0,809
A4HXY2	1fot.1.A	0,74	-2,7	0,95	50	2,80A	None	pocket 1	0,765
	4wb8.1.A	0,77	-0,56	0,95	51,27	1,55A	ATP - MG	pocket 1	0,737
	4wb5.1.A	0,77	-0,51	0,95	51,43	1,64A	ATP - MG	pocket 2	0,809
E9BDT9	1fot.1.A	0,74	-2,7	0,95	50	2,80A	None	pocket 1	0,765

Table S4. Summary of the modelling variables and the druggability score for the ATM kinases detected in the five species of *Leishmania* analysed.

UniProt ID	PDB template	GMQE	QMEAN	COV	ID	RES	Ligands	fpocket	DS
A4H3A6	4jsn.1.A	0,03	-4,28	0,12	23,54	3,20A	None	pocket 2	0,509
E9AC86	4jsn.1.A	0,03	-3,59	0,11	23,77	3,20A	None	pocket 1	0,825
E9AJH9	4jsn.1.A	0,03	-3,63	0,11	23,71	3,20A	None	pocket 11	0,865
A4HRL5	4jsp.1.A	0,03	-4,59	0,11	24,13	3.30A	AGS - MG	pocket 4	0,765
E9B7F7	4jsn.1.A	0,03	-4,15	0,11	24,09	3,20A	None	pocket 1	0,587

Table S5. Summary of the modelling variables and the druggability score for the AMPK kinases detected in the five species of *Leishmania* analysed.

UniProt ID	PDB template	GMQE	QMEAN	COV	ID	RES	Ligands	fpocket	DS
	5ezv.1.A	0,31	-0,96	0,4	52,94	2,99A	C1V – C2Z - STU	pocket 1	0,57
A4HHK1	2hak.6.A	0,27	-2,72	0,39	40,82	2,60A	None	pocket 6	0,821
	5ezv.1.A	0,31	-0,85	0,4	53,4	2,99A	C1V – C2Z - STU	pocket 2	0,563
E9AE64	5iso.1.A	0,3	-1,47	0,4	53,73	2,63A	AMP - STU - 992	pocket 1	0,485
E9ALM1	6b1u.2.A	0,31	-1,39	0,4	52,94	2,77A	AMP- CG7 - STU	pocket 1	0,725

	2hak.6.A	0,27	-2,79	0,39	41,64	2,60A	None	pocket 6	0,576
	4ynz.1.B	0,26	-2,01	0,39	41,77	2,00A	None	pocket 5	0,524
	2hak.6.A	0,26	-2,86	0,39	41,77	2,60A	None	pocket 6	0,526
A4I4Q9	6b1u.2.A	0,31	-1,4	0,4	53,25	2,77A	IMD – AMP – CG7 - STU	pocket 1	0,725
	6b1u.2.A	0,31	-1,4	0,4	53,25	2,77A	IMD – AMP – CG7 - STU	pocket 1	0,725
	5ezv.1.A	0,3	-1,76	0,4	53,25	2,99A	C1V – C2Z - STU	pocket 1	0,576
E9BL11	4yom.1.A	0,27	-2,52	0,39	38,32	2,49A	EDO	pocket 2	0,55

Table S6. Summary of the modelling variables and the druggability score for the mTOR kinases detected in the five species of *Leishmania* analysed.

UniProt ID	PDB template	GMQE	QMEAN	COV	ID	RES	Ligands	fpocket	DS
	4jsn.1.A	0,29	-3,73	0,44	38,33	3,20A	None	pocket 1	0,465
A4HBF9	5wbu.1.A	0,29	-3,71	0,44	36,3	3,42A	None	pocket 49	0,742
Q4Q0C8	4jsn.1.A	0,26	-3,71	0,42	38,39	3,20A	None	pocket 9	0,532
E9AU66	4jsn.1.A	0,26	-3,73	0,42	38,48	3,20A	None	pocket 1	0,643
A4IE36	4jsn.1.A	0,25	-3,72	0,42	38,42	3,20A	None	pocket 11	0,658
E9BV14	4jsn.1.A	0,26	-3,71	0,42	38,33	3,20A	None	pocket 9	0,584

Table S7. Summary of the modelling variables and the druggability score for the GSK3 kinases detected in the five species of *Leishmania* analysed.

UniProt ID	PDB template	GMQE	QMEAN	COV	ID	RES	Ligands	fpocket	DS
	3e3p.1.A	0,94	-0,58	1	93,52	2,00A	None	pocket 2	0,625
A4H9D1	3sd0.1.B	0,72	-2,72	0,9	50,16	2,70A	EPE - TSK	pocket 1	0,606
	3e3p.1.A	0,97	-0,25	1	100	2,00A	None	pocket 2	0,658
Q4QE15	3sd0.1.B	0,72	-1,81	0,9	49,53	2,70A	EPE - TSK	pocket 1	0,628
	3e3p.1.A	0,96	-0,61	1	96,9	2,00A	None	pocket 3	0,53
E9ARG4	3sd0.1.B	0,72	-2,13	0,9	49,53	2,70A	EPE - TSK	pocket 2	0,436
	3e3p.1.A	0,97	-0,41	1	98,03	2,00A	None	pocket 2	0,658
A4HXQ3	5f94.1.A	0,73	-2,82	0,91	49,23	2,51A	3UO	pocket 1	0,788
	3e3p.1.A	0,97	-0,43	1	98,31	2,00A	None	pocket 2	0,658
E9BDK8	3sd0.1.A	0,72	-2,18	0,9	49,84	2,70A	EPE - TSK	pocket 1	0,579

Table S8. Summary of the modelling variables and the druggability score for the PI3K kinases detected in the five species of *Leishmania* analysed.

**UniProt ID**	**PDB template**	**GMQE**	**QMEAN**	**COV**	**ID**	**RES**	**Ligands**	**fpocket**	**DS**
A4HDR3	5dfz.1.B	0,47	-5,83	0,71	27,6	4,40A	None	pocket 1	0,837
	6c1s.1.A	0,08	-3,4	0,2	25,29	2,31A	EFV - SO4	pocket 1	0,836
E9AE04	4bfr.1.A	0,08	-3,16	0,19	23,64	2,80A	J82	pocket 5	0,818
	4l1b.1.A	0,63	-3,92	0,92	32,14	2,59A	SO4	pocket 35	0,565
E9APJ6	4bfr.1.A	0,61	-4,05	0,93	32,68	2,80A	J82	pocket 1	0,534
A4HVU4	6eyz.1.A	0,58	-3,96	0,88	31,31	2,20A	C5W	pocket 17	0,92
E9BBN7	5ae9.1.A	0,56	-3,72	0,84	28,17	2,44A	OKO	pocket 17	0,53

Table S9. List of best 20 compounds per kinase model based on the AutoDock Vina score [Attached xlsx file].

Table S10. Full list of compounds IDs and SMILES representations of their chemical structures. [Attached xlsx file].

Supplementary Text

S1. Details of other proteins detected from the PI3K/AKT pathway mapping

S1.1 14-3-3

The highly conserved, multifunctional phospho-serine/phospho-threonine binding proteins 14-3-3 have two roles in apoptosis: increases the tumour suppressor p53 activity to induce the transcription of pro-apoptotic genes such as *bad* [1] but also alter pro-apoptotic proteins (BAD, Bax, FOXO and Ask1) subcellular location to block their activation [2]. 14-3-3 proteins act as chaperones also for other proteins like Raf-1, involved in cell proliferation and survival [2]. Several small molecules identified as 14-3-3 inhibitors, reviewed in [1], have been assayed as drug candidates in cancer therapy. Recently, a novel series of specific sphingosine-like compounds able to induce mitochondrial apoptosis *in vitro* and reduce tumour growth *in vivo* by disrupting 14-3-3 dimerization has been reported [3].

S1.2 Eukaryotic translation initiation factor- eIF4E

In eukaryotes initiation of cap-dependent translation relies on the small ribosome subunit recruitment to the 5 –end of the mRNA (denoted cap4 in trypanosomatids) which is facilitated by eIF4F complex. eIF4F consists of a small cap-binding protein, eIF4E, an ATP-dependent RNA helicase, eIF4A, and a large scaffold protein, eIF4G. In their hypophosphorylated state, 4E-BPs act as competitive inhibitors of eIF4F complex formation. When activated through PI3K/AKT pathway, mTOR phosphorylates 4E-BP and eIF4G, releasing eIF4E, allowing for eIF4F complex formation and therefore increasing cap dependent translation. In the field of cancer, eIF4F complex has been proposed as a therapeutic target because growth-related and anti-apoptotic proteins are encoded by

structurally complex mRNAs which have a high requirement for eIF4F[4]. Compounds that impede eIF4E–eIF4G complex formation (4EGI-1) suppressed the growth and induced apoptosis in several types of cancer[5].

S1.3 Heat Shock Protein-Hsp90

Inhibition of the molecular chaperone Hsp90 induces cell cycle arrest and apoptosis as it causes the simultaneous degradation of different client proteins involved in all levels of cell signalling [6]. Hsp90 and other chaperones function as survival factors, particularly under stress conditions, e.g., prevents procaspase-9 recruitment to the apoptosome by binding Apaf-1 [7]; stabilizes and protects AKT from phosphatase 2A (PP2A) dephosphorylation, hence inhibiting apoptosis [8].

S1.4 C-myb

C-myb is a highly conserved DNA binding transcriptional regulator that controls the expression of certain genes related to cell proliferation, differentiation and development [9]. C-myb also activates the expression of pro-survival proteins such as Bcl-X_L [10,11]. There are studies reporting c-myb disruption as an inductor of apoptosis in tumorigenic cells by inhibiting AKT pathway activation [12].

S1.5 JAK

The Janus kinase (JAK) is the physiological activator of the signal transducer and activator of transcription 3 (STAT3). When activated, STAT3 may play a role in apoptosis by increasing the expression of proteins like survivin, a member of the Inhibitors of Apoptosis (IAP) or repressing the expression of the pro-apoptotic factor p53 that up-regulates proteins like APAF1, caspase 6 and FAS [13]. Specific inhibition of JAK2 (i.e.: AG490 or AZD1480) has been correlated with increase in apoptotis as associated with lower expression of the proteins Bcl-XL and Bcl-2 [14] [15]. More importantly, there are JAK inhibitors already approved for human use (Ruxolitinib and Tofacitinib) and many others are undergoing clinical trials.

S1.6 NF-κB

Nuclear factor of κB (NF-κB) is a family of transcription factors activators of several genes that promote cell proliferation (cyclins and growth factors) or block programmed cell death (cIAPS, cFLIP, Bfl-1, Bcl-2, and Bcl-xl) [16]. Numerous inhibitors of NF-κB have shown to block proliferation or render tumour cells more sensitive to apoptosis-inducing agents, however none has yet been approved for human use [17].

S1.7 S6 kinase

S6 serine/threonine kinase is a downstream effector of mTORC1 involved in protein synthesis and cell growth. Because S6K is considered a `junctional molecule´, that is, it's a node between different signal transduction pathways, it is thought to constitute an ideal target for cancer therapy [18] but we could only find a limited number studies relating S6K and apoptosis [19,20] including one clinical phase I trial evaluating an inhibitor of S6K [21].

S1.8 PHLPP and PTEN

The protein phosphatase PHLPP and the lipid phosphatase PTEN conform a tumour suppressor with synergistic effects on the control of the PI3K/AKT cascade [22]. PHLPP mediates dephosphorylation of AKT and S6 kinase, therefore promoting apoptosis, decreasing proliferation and depleting overall protein synthesis [23]. Thus, PHLPP is a potential therapeutic target to suppress oncogenic pathways [23]. On the other hand, the major function of PTEN is dephosphorylation of PtdIns (3,4,5) regulator of AKT activation and the main product of PI3K thus antagonizing its biological action [24]. In addition, promising studies *in vitro* have identified several regulatory mechanisms to modulate PTEN activity [24]. Interestingly, cell migration, invasion and adhesion have been listed as other functions of PTEN [25]. We found no studies reporting either of these proteins in *Leishmania*.

S2. Role of other proteins detected in the PI3K/AKT homologue pathway in *Leishmania* spp.

Regarding other proteins within the PI3K/AKT survival pathway, we found that 14-3-3 was recently reported in *L. infantum* and is thought to be a virulence factor for prolonging the life

span of the host cell [26]. A study has linked 14-3-3 with drug-induced cell death in *Leishmania* [27].

As for eIF4E, four isoforms have been described in *Leishmania*, denoted LeishIF4E-1 through LeishIF4E-4. Nonetheless, little is known of their biological functions. LeishIF4E-1 would be functional during thermal stress [28], LeishIF4E-3 is located in stress granules together with inactive mRNAs during nutritional deprivation and LeishIF4E-4 complex engages in the translation initiation process in promastigotes [28]. Hsp90 (a.k.a Hsp83) is one of the most abundant proteins in *Leishmania*. In *L. donovani,* the Hsp90 complex has an essential role in drug resistance, proliferation and survival in both extra and intracellular forms of the parasite [29]. Pharmacological inhibition of *Leishmania* Hsp90 with specific inhibitors as geldanamycin, radiciol [30] and 17-AAG [31] affects transformation of promastigotes into amastigotes and halts intracellular proliferation of amastigotes in *ex vivo* macrophages. The binding motif to Hsp90, PxxP is conserved in *Leishmania* AKT-*like* AGC domain and probably the molecular mechanism underlying both proteins interaction and function is conserved in *Leishmania*.

It is important to clarify that some of these proteins associated to the PI3K-AKT pathway have not being previously reported as *Leishmania* homologous. For example, a myb-*like* domain has been described as part of telomere binding proteins in *L. amazonensis,* but no c-myb [32]. In our data, c-myb is exclusively present in *L. braziliensis.* Other one is NF-κB, which had not been described in *Leishmania* but was detected by our method. To the best of our knowledge, none of the several thousand of the existing NF-κB inhibitors have been evaluated as potential anti-*Leishmania* compounds. S6K1 had not been previously reported in *Leishmania* but its major substrate, ribosomal S6 component of the 40S subunit, was cloned in *L. infantum* (LiS6). LiS6 sequence shows common features with that in eukaryotes but differs in key regulatory phosphorylation sites which might affect its function in the parasite [33].

S3. Apoptosis-*like* potential effectors in *Leishmania* spp.

Except for Raf, we found three out of four members of the Ras/Raf-MEK-/MAPK-ERK pathway. This signal transduction pathway is present in all eukaryotes, regulates the gene expression and activity of many proteins involved in control of cell division, growth, proliferation or apoptosis. A putative MEK (named MRK1 for MEK-related kinase) was identified in *L .major* [34] and at least 15 putative genes for MAPKs are codified in the *L. major, L. mexicana, L. infantum* y *L. braziliensis* genome [35]; these are required for flagellar development, intracellular survival and viability [36]. As for the Ras or Rho subfamilies though present, are highly divergent [37]. Mitogenic Ras/MAPK and survival PI3K/AKT pathways overlap at several nodes (being BAD an essential one [38]) determining cell fate, as a result, their simultaneous blockade it is being explored in cancer [39]. These cascades regulate each other in a context-dependent manner via cross-inhibition (with the involvement of *Leishmania* homologs 14-3-3, PP2A or AKT) and cross-activation. More details on this topic can be found in [40,41]. Due to their reciprocal regulation, and the activation of compensatory signaling, Ras/MAPK stands as an important pathway when considering apoptosis induction as leishmaniasis therapy.

In metazoans, mitochondria dysfunction is a main activator of apoptosis. During intrinsic cell death Bcl-2 members are responsible for mitochondrial outer membrane permeabilization, hence releasing pro-apoptotic factors, like cytochrome C into the cytosol. To the best of our knowledge none of the pro-apoptotic Bcl-2 members BAX, BAD, BAK, or BID have been reported in *Leishmania*, and were not retrieved in our *in silico* search. However, functional homologous of Bax might be present in *Leishmania* [42]. On the other hand, although we did not find BAD, we retrieved its inhibitors AKT, 14-3-3 and S6K1 and its activator, calpain, while other studies further support the existence of BH3 bearing proteins [43]. Pro-apoptotic cytochrome c binds in the cytosol to several molecules of Apaf-1; this protein complex known as apoptosome activates the initiator caspase 9. Several species of *Leishmania* are known to secrete cytochrome c upon apoptotic stimuli [44]. A homologue of Apaf-1 protein has been previously described *in silico* [45]; we further identified homologues in *L. major, L, braziliensis*, and *L. donovani.*

References

1 Aghazadeh Y, Papadopoulos V. The Role of the 14-3-3 Protein Family in Health, Disease, and Drug Development. *Drug Discov. Today* 2016; **21**: 278–287; DOI:10.1016/j.drudis.2015.09.012.

2 Muslin AJ, Xing H. 14-3-3 Proteins: Regulation of Subcellular Localization by Molecular Interference. *Cell. Signal.* 2000; **12**: 703–709.

3 Woodcock JM, Coolen C, Goodwin KL, Baek DJ, Bittman R, Samuel MS, Pitson SM, Lopez AF. Destabilisation of Dimeric 14-3-3 Proteins as a Novel Approach to Anti-Cancer Therapeutics. *Oncotarget* 2015; **6**: 14522–14536; DOI:10.18632/oncotarget.3995.

4 Walsh D. Manipulation of the Host Translation Initiation Complex EIF4F by DNA Viruses. *Biochem. Soc. Trans.* 2010; **38**: 1511–1516; DOI:10.1042/BST0381511.

5 Siddiqui N, Sonenberg N. Signalling to EIF4E in Cancer. *Biochem. Soc. Trans.* 2015; **43**.

6 Maloney A, Workman P. HSP90 as a New Therapeutic Target for Cancer Therapy: The Story Unfolds. *Expert Opin. Biol. Ther.* 2002; **2**: 3–24; DOI:10.1517/14712598.2.1.3.

7 Kurokawa M, Zhao C, Reya T, Kornbluth S. Inhibition of Apoptosome Formation by Suppression of Hsp90beta Phosphorylation in Tyrosine Kinase-Induced Leukemias. *Mol. Cell. Biol.* 2008; **28**: 5494–5506; DOI:10.1128/MCB.00265-08.

8 Sato S, Fujita N, Tsuruo T. Modulation of Akt Kinase Activity by Binding to Hsp90. *Proc. Natl. Acad. Sci. U. S. A.* 2000; **97**: 10832–10837; DOI:10.1073/pnas.170276797.

9 Oh I-H, Reddy EP. The Myb Gene Family in Cell Growth, Differentiation and Apoptosis. *Oncogene* 1999; **18**: 3017–3033; DOI:10.1038/sj.onc.1202839.

10 Huang Y, Jiang Y, Lu W, Zhang Y. Nemo-like Kinase Associated with Proliferation and Apoptosis by c-Myb Degradation in Breast Cancer. *PLoS One* 2013; **8**: e69148; DOI:10.1371/journal.pone.0069148.

11 Ramsay RG, Barton AL, Gonda TJ. Targeting C-Myb Expression in Human Disease. *Expert Opin. Ther. Targets* 2003; **7**: 235–248; DOI:10.1517/14728222.7.2.235.

12 Kim SY, Yang YS, Hong KH, Jang KY, Chung MJ, Lee DY, Lee JC, Yi HK, Nam S-Y, Hwang PH. Adenovirus-Mediated Expression of Dominant Negative c-Myb

Induces Apoptosis in Head and Neck Cancer Cells and Inhibits Tumor Growth in Animal Model. *Oral Oncol.* 2008; **44**: 383–392; DOI:10.1016/j.oraloncology.2007.05.004.

13 Al Zaid Siddiquee K, Turkson J. STAT3 as a Target for Inducing Apoptosis in Solid and Hematological Tumors. *Cell Res.* 2008; **18**: 254–267; DOI:10.1038/cr.2008.18.

14 Xiong H, Zhang Z-G, Tian X-Q, Sun D-F, Liang Q-C, Zhang Y-J, Lu R, Chen Y-X, Fang J-Y. Inhibition of JAK1, 2/STAT3 Signaling Induces Apoptosis, Cell Cycle Arrest, and Reduces Tumor Cell Invasion in Colorectal Cancer Cells. *Neoplasia* 2008; **10**: 287–297.

15 Stuart E, Buchert M, Putoczki T, Thiem S, Farid R, Elzer J, Huszar D, Waring PM, Phesse TJ, Ernst M. Therapeutic Inhibition of Jak Activity Inhibits Progression of Gastrointestinal Tumors in Mice. *Mol. Cancer Ther.* 2014; **13**: 468–474; DOI:10.1158/1535-7163.MCT-13-0583-T.

16 Karin M, Cao Y, Greten FR, Li Z-W. NF-KappaB in Cancer: From Innocent Bystander to Major Culprit. *Nat. Rev. Cancer* 2002; **2**: 301–310; DOI:10.1038/nrc780.

17 Gupta SC, Sundaram C, Reuter S, Aggarwal BB. Inhibiting NF-KB Activation by Small Molecules as a Therapeutic Strategy. *Biochim. Biophys. Acta* **1799**: 775–787; DOI:10.1016/j.bbagrm.2010.05.004.

18 Zhao XF, Gartenhaus RB. Phospho-P70S6K and Cdc2/Cdk1 as Therapeutic Targets for Diffuse Large B-Cell Lymphoma. *Expert Opin. Ther. Targets* 2009; **13**: 1085–1093; DOI:10.1517/14728220903103833.

19 Choi H-N, Jin H-O, Kim J-H, Hong S-E, Kim H-A, Kim E-K, Lee JK, Park I-C, Noh WC. *Inhibition of S6K1 Enhances Glucose Deprivation-Induced Cell Death via Downregulation of Anti-Apoptotic Proteins in MCF-7 Breast Cancer Cells*, Vol. 432. 2013; DOI:10.1016/j.bbrc.2013.01.074.

20 Sridharan S, Basu A. S6 Kinase 2 Promotes Breast Cancer Cell Survival via Akt. *Cancer Res.* 2011; **71**: 2590–2599; DOI:10.1158/0008-5472.CAN-10-3253.

21 Qiu Z-X, Sun R-F, Mo X-M, Li W-M. The P70S6K Specific Inhibitor PF-4708671 Impedes Non-Small Cell Lung Cancer Growth. *PLoS One* 2016; **11**: e0147185; DOI:10.1371/journal.pone.0147185.

22 Molina JR, Agarwal NK, Morales FC, Hayashi Y, Aldape KD, Cote G, Georgescu M-M. PTEN, NHERF1 and PHLPP Form a Tumor Suppressor Network That Is Disabled in Glioblastoma. *Oncogene* 2012; **31**: 1264–1274; DOI:10.1038/onc.2011.324.

23 Newton AC, Trotman LC. Turning off AKT: PHLPP as a Drug Target. *Annu. Rev. Pharmacol. Toxicol.* 2014; **54**: 537–558; DOI:10.1146/annurev-pharmtox-011112-140338.

24 Chalhoub N, Baker SJ. PTEN and the PI3-Kinase Pathway in Cancer. *Annu. Rev. Pathol.* 2009; **4**: 127–150; DOI:10.1146/annurev.pathol.4.110807.092311.

25 Yamada KM, Araki M. Tumor Suppressor PTEN: Modulator of Cell Signaling, Growth, Migration and Apoptosis. *J. Cell Sci.* 2001; **114**: 2375–2382.

26 da Fonseca Pires S, Fialho LC, Silva SO, Melo MN, de Souza CC, Tafuri WL, Bruna Romero O, de Andrade HM. Identification of Virulence Factors in Leishmania Infantum Strains by a Proteomic Approach. *J. Proteome Res.* 2014; **13**: 1860–1872; DOI:10.1021/pr400923g.

27 Vergnes B, Gourbal B, Girard I, Sundar S, Drummelsmith J, Ouellette M. A Proteomics Screen Implicates HSP83 and a Small Kinetoplastid Calpain-Related Protein in Drug Resistance in Leishmania Donovani Clinical Field Isolates by Modulating Drug-Induced Programmed Cell Death. *Mol. Cell. Proteomics* 2006; **6**: 88–101; DOI:10.1074/mcp.M600319-MCP200.

28 Zinoviev A, Manor S, Shapira M. Nutritional Stress Affects an Atypical Cap-Binding Protein in Leishmania. *RNA Biol.* 2012; **9**: 1450–1460; DOI:10.4161/rna.22709.

29 Hombach A, Clos J. No Stress--Hsp90 and Signal Transduction in Leishmania. *Parasitology* 2014; **141**: 1156–1166; DOI:10.1017/S0031182013002151.

30 Wiesgigl M, Clos J. Heat Shock Protein 90 Homeostasis Controls Stage Differentiation in Leishmania Donovani. *Mol. Biol. Cell* 2001; **12**: 3307–3316; DOI:10.1091/MBC.12.11.3307.

31 Petersen AL de OA, Guedes CES, Versoza CL, Lima JGB, de Freitas LAR, Borges VM, Veras PST, Desjeux P, Kedzierski L, Zhu Y, et al.. 17-AAG Kills Intracellular Leishmania Amazonensis While Reducing Inflammatory Responses in Infected

Macrophages. *PLoS One* 2012; **7**: e49496; DOI:10.1371/journal.pone.0049496.

32 Lira CBB, de Siqueira Neto JL, Khater L, Cagliari TC, Peroni LA, dos Reis JRR, Ramos CHI, Cano MIN. LaTBP1: A Leishmania Amazonensis DNA-Binding Protein That Associates in Vivo with Telomeres and GT-Rich DNA Using a Myb-like Domain. *Arch. Biochem. Biophys.* 2007; **465**: 399–409; DOI:10.1016/j.abb.2007.06.020.

33 Gonzalez-Aseguinolaza G, Taladriz S, Marquet A, Larraga V. Cloning and Structural Analysis of the Gene Encoding the Ribosomal Protein S6 from the Parasite Leishmania Infantum. *Biochem. Biophys. Res. Commun.* 1998; **248**: 464–468; DOI:10.1006/bbrc.1998.8927.

34 Agron PG, Reed SL, Engel JN. An Essential, Putative MEK Kinase of Leishmania Major. *Mol. Biochem. Parasitol.* 2005; **142**: 121–125; DOI:10.1016/j.molbiopara.2005.03.007.

35 Wiese M. Leishmania MAP Kinases--Familiar Proteins in an Unusual Context. *Int. J. Parasitol.* 2007; **37**: 1053–1062; DOI:10.1016/j.ijpara.2007.04.008.

36 Cayla M, Rachidi N, Leclercq O, Schmidt-Arras D, Rosenqvist H, Wiese M, Späth GF. Transgenic Analysis of the Leishmania MAP Kinase MPK10 Reveals an Auto-Inhibitory Mechanism Crucial for Stage-Regulated Activity and Parasite Viability. *PLoS Pathog.* 2014; **10**: e1004347; DOI:10.1371/journal.ppat.1004347.

37 Field MC. Signalling the Genome: The Ras-like Small GTPase Family of Trypanosomatids. *Trends Parasitol.* 2005; **21**: 447–450; DOI:10.1016/j.pt.2005.08.008.

38 Fang X, Yu S, Eder A, Mao M, Bast RC, Boyd D, Mills GB. Regulation of BAD Phosphorylation at Serine 112 by the Ras-Mitogen-Activated Protein Kinase Pathway. *Oncogene* 1999; **18**: 6635–6640; DOI:10.1038/sj.onc.1203076.

39 Chappell WH, Steelman LS, Long JM, Kempf RC, Abrams SL, Franklin RA, Bäsecke J, Stivala F, Donia M, Fagone P, Malaponte G, Mazzarino MC, Nicoletti F, Libra M, Maksimovic-Ivanic D, Mijatovic S, Montalto G, Cervello M, Laidler P, Milella M, Tafuri A, Bonati A, Evangelisti C, Cocco L, Martelli AM, McCubrey JA. Ras/Raf/MEK/ERK and PI3K/PTEN/Akt/MTOR Inhibitors: Rationale and Importance to Inhibiting These Pathways in Human Health. *Oncotarget* 2011; **2**:

135–164; DOI:10.18632/oncotarget.240.

40 Aksamitiene E, Kiyatkin A, Kholodenko BN. Cross-Talk between Mitogenic Ras/MAPK and Survival PI3K/Akt Pathways: A Fine Balance. *Biochem. Soc. Trans.* 2012; **40**: 139–146; DOI:10.1042/BST20110609.

41 Mendoza MC, Er EE, Blenis J. The Ras-ERK and PI3K-MTOR Pathways: Cross-Talk and Compensation. *Trends Biochem. Sci.* 2011; **36**: 320–328; DOI:10.1016/j.tibs.2011.03.006.

42 Arnoult D, Akarid K, Grodet A, Petit PX, Estaquier J, Ameisen JC. On the Evolution of Programmed Cell Death: Apoptosis of the Unicellular Eukaryote Leishmania Major Involves Cysteine Proteinase Activation and Mitochondrion Permeabilization. *Cell Death Differ.* 2002; **9**: 65–81; DOI:10.1038/sj.cdd.4400951.

43 Alzate JF, Arias A, Mollinedo F, Rico E, de la Iglesia-Vicente J, Jiménez-Ruiz A. Edelfosine Induces an Apoptotic Process in Leishmania Infantum That Is Regulated by the Ectopic Expression of Bcl-XL and Hrk. *Antimicrob. Agents Chemother.* 2008; **52**: 3779–3782; DOI:10.1128/AAC.01665-07.

44 Jiménez-Ruiz A, Alzate J, MacLeod E, Lüder CG, Fasel N, Hurd H, Kroemer G, Galluzzi L, Vandenabeele P, Abrams J, Alnemri E,et. al. Apoptotic Markers in Protozoan Parasites. *Parasit. Vectors* 2010; **3**: 104; DOI:10.1186/1756-3305-3-104.

45 Gannavaram S, Debrabant A. Programmed Cell Death in Leishmania: Biochemical Evidence and Role in Parasite Infectivity. *Front. Cell. Infect. Microbiol* 2012.
